# Coping with the COVID-19 pandemic: Contemplative practice behaviors are associated with better mental health outcomes and compliance with shelter-in-place orders in a prospective cohort study

**DOI:** 10.1016/j.pmedr.2021.101451

**Published:** 2021-06-12

**Authors:** Benjamin W. Chrisinger, Tia Rich, David Lounsbury, Katy Peng, Janice Zhang, Catherine A. Heaney, Ying Lu, Ann W. Hsing

**Affiliations:** a University of Oxford, Department of Social Policy and Intervention, United Kingdom; b Stanford Prevention Research Center, Department of Medicine, Stanford School of Medicine, Stanford University, Stanford, CA 94035, United States; c Department of Epidemiology and Population Health, Albert Einstein College of Medicine, Bronx, NY 10461, United States; d Department of Psychology, Stanford University, Stanford, CA 94305, United States; e Department of Biomedical Data Science, Stanford School of Medicine, Stanford University, Stanford, CA 94305, United States; f Department of Epidemiology and Population Health, Stanford School of Medicine, Stanford University, Stanford, CA 94305, United States; g Stanford Cancer Institute, Stanford School of Medicine, Stanford University, Stanford, CA 94305, United States

**Keywords:** COVID-19, Contemplative practices, Meditation, Compassion, Psychosocial, Stress, Resilience, Emotions, Distress

## Abstract

Psychosocial health can influence the development and experience of several chronic diseases, and has been negatively affected for many individuals amid the COVID-19 global pandemic. To understand the impact of contemplative practices on emotional and mental health during COVID-19, the Stanford WELL for Life Study (US component), incorporated a series of additional surveys into its ongoing study. A total of 1,097 participants residing in California who responded to at least one of three COVID-19 surveys were included in this analysis. Linear and generalized mixed-effects regression models were used to investigate relationships between individual contemplative practice behaviors (CPB) (embodied observing meditation, non-reactive mindfulness meditation, self-compassion cultivation, cultivation of compassion for others) and four psychosocial outcomes measured in the original WELL questionnaire (resilience, dealing with stress, positive emotions, and negative emotions). In addition, the associations between CPB and depression, distress, and compliance with local Shelter-In-Place orders were also investigated. Participants who engaged in any contemplative practice reported significantly more resilience and positive emotions, dealing better with stress, lower distress, and were less likely to report an experience with depression in the last week. Similar findings held when CPB was modeled as a continuous variable. Significant interactions between the duration of the SIP and CPB were also observed for resilience and SIP compliance outcomes, indicating that steeper declines were observed among participants with little or no CPB across the study period. Further investigation into the potential protective benefits of CPB during times of major disruption and uncertainty is warranted.

## 1 Introduction

A growing body of research from populations around the world suggests that a major consequence of the continuing COVID-19 pandemic is widespread mental health burden, including increased stress, distress, anxiety, depression, and problematic substance use (El-Hage et al., 2020, Huang and Zhao, 2020, Marton et al., 2020, Rossell et al., 2021, Daly et al., 2021, Charles et al., 2021, Castellano-Tejedor et al., 2021, Luo et al., 2020). More than fifty years of research has also shown that contemplative practices can support mental health and emotional well-being, reduce stress, and cope with or prevent illness (Goleman and Davidson, 2017).

Contemplative practices are “efforts that promote human flourishing by training the mind” (Dahl and Davidson, 2019), and are intended to cultivate: 1) self-knowledge and insight, including self-soothing and emotion modulation skills (Kabat-Zinn, 2013), 2) prosocial thoughts and behavior, and 3) purpose and meaning in life. Contemplative practices also foster cognitive, affective, and conceptual states, traits and processes (Dorjee, 2016). By cultivating relaxation and calm within one’s body, contemplative practices can mitigate adverse biological (e.g., elevated cortisol or blood pressure) and behavioral (e.g., substance abuse) responses, and stress-related medical conditions (e.g., heart disease, gastrointestinal disorders, depression) (Lazar et al., 2000). Contemplative practices also can broaden perspective, reduce intrusive rumination, raise one’s awareness, cultivate insights regarding unresolved emotional and stressful situational issues, and enhance positive emotions (Sprecher and Fehr, 2006), emotion self-modulation skills, interpersonal effectiveness and posttraumatic growth (i.e., positive change resulting from highly challenging life events) (Tedeschi and Blevins, 2015).

Contemplative practices have also been identified as having physical and psychosocial health benefits, including reducing stress (Grossman et al., 2004). However, most contemplative practices intervention studies have not focused on the general population; rather, they have often been clinically-based treatments for patients with diseases or psychological disorders (Goldberg et al., 2018); including treatment of cardiovascular disease (Abbott et al., 2014, Levine et al., 2017, Scott-Sheldon et al., 2020); depression (Gilbert, 2005, Blanck et al., 2018); anxiety (Gilbert, 2005, Blanck et al., 2018); and post-traumatic stress disorder (Cushing and Braun, 2018). Furthermore, there is little research on the role of contemplative practices behaviors prior in promoting positive emotions, physical and emotional health, and resilience during and following a stressful life event, and supporting subsequent posttraumatic growth (Prati and Pietrantoni, 2009).

In light of the COVID-19 pandemic, important questions emerge regarding the ability of contemplative practices to mitigate the negative mental health effects that have been observed (El-Hage et al., 2020, Huang and Zhao, 2020, Marton et al., 2020, Rossell et al., 2021, Daly et al., 2021, Charles et al., 2021, Castellano-Tejedor et al., 2021). Health officials have faced a mounting challenge as “compliance fatigue” has emerged among a public struggling with social distancing guidance, including orders to reduce or eliminate trips outside of the home which have been shown to both dramatically reduce social contacts and present additional mental health concerns (Daly et al., 2021, Castellano-Tejedor et al., 2021, Barari et al., 2020, Armbruster and Klotzbücher, 2020). While global vaccination campaigns against COVID-19 get underway, orders to remain at home and socially distanced remain the primary non-pharmacological interventions available to policymakers wishing to slow the spread of the virus (Cowling et al., 2020).

This study investigates whether contemplative practices before and during the COVID-19 pandemic are associated with resilience, dealing with stress, emotions (positive and negative), distress, and depression, and hypothesize that contemplative practitioners experienced less of these mental health burdens. Furthermore, we examine the relationship between compliance with local public health “Stay at Home” orders and contemplative practices. We hypothesize that contemplative practitioners may be better able to cope with the destabilizing effects of the pandemic and local Stay at Home orders. More broadly, this study aims to provide further evidence on the possible mental health benefits of contemplative practices that could be investigated in general population interventions or other mechanistic studies that examine these behaviors alongside other lifestyle behaviors during the pandemic.

## 2 Methods

### 2.1 Survey design

The Stanford WELL for Life Study (“WELL”) (US component) was initiated in 2016 as a prospective data registry focusing on health and well-being in the San Francisco Bay Area. Participants were continuously recruited through community engagements and through electronic advertising and communications; additional recruitment description has been published elsewhere (Heaney et al., 2017). The online WELL questionnaire (W0) included a multi-dimensional measure of well-being containing ten conceptual domains, as well as sociodemographic variables and self-reports of medical history.

As the COVID-19 pandemic was emerging in the United States, follow-up surveys (“COVID-19 surveys”) were rapidly developed and incorporated into the ongoing WELL Study. Following the implementation of a Shelter-in-Place (SIP) order in the five San Francisco Bay Area counties on March 17, 2020, and across all of California on March 20, 2020, individuals were required by law to remain at home, except for a small set of “essential” trips for food, prescriptions, and health care, or for work in a “critical infrastructure sector.” The COVID-19 surveys were designed to capture individual experiences during COVID-19 and the SIP order. The first COVID-19 survey was administered on March 23, 2020 (T0), a second survey (follow up) was launched on April 3, 2020 (10-day survey, T1), and a third on April 23, 2020 (1-month survey, T2). As of April 23, 2020, 1,244 of the original WELL participants have responded to at least one COVID-19 survey, with over 95% of these participants responding to all survey items.

Only participants residing in California (n = 1,097) were included in this CPB analysis. Additionally, WELL questionnaire data (W0) that were collected more than a year before the WELL-COVID study was initiated were excluded. The primary WELL Study and the COVID-19 follow-up Study (COVID-19 WELL) were approved by the Stanford Medicine Institutional Review Board.

### 2.2 Primary independent variables

The primary independent variables of interest are the frequency of performing four kinds of contemplative practices related to mindfulness and compassion, measured with four questions in the WELL survey. These questions were asked at W0, T1 and T2. Practices of mindfulness were measured by frequency over the last two weeks of *embodied-observing practices* (breathing deeply, gently stretching, noticing your senses) and by *non-reactive practices* (observing emotions and thoughts as they arise rather than being caught up in them). Compassion practices were measured by the frequency of *self-compassion practices* (pausing routine activities to observe and modify the way one is thinking to offer more compassion, love or kindness to oneself) and *compassion practices toward others (*pausing routine activities to observe and modify the way one is thinking to offer more compassion, love or kindness toward others). The frequency of each set of practices was measured on an ordinal scale (0–4: Never, Almost never, Sometimes, Fairly often, Very often). For each individual, a composite measure, CPB, was calculated as the mean of the four contemplative practice questions. Two representations of CPB are used in this study: A) Presence of any contemplative practices (binary, responding “Sometimes” or more frequent to any of the four contemplative practice questions at the time point)*;* and B) CPB (continuous, average frequency of the four contemplative practices at the time point). See Supplemental Table 1 for a summary of the CPB questions and variables.

### 2.3 Primary outcomes

Seven outcomes were investigated relating to potential individual psychosocial and behavioral effects during the COVID-19 study. Four continuous outcomes measured at T0, T1, T2 were derived from the initial WELL questionnaire (W0), which includes questions that characterize ten different domains of well-being (Heaney et al., 2017): dealing with stress (five-question construct adapted from the Perceived Stress Scale (Cohen et al., 1983); higher scores indicate dealing better with stress), resilience (nine-question construct adapted from the Connor-Davidson Resilience Scale (Connor and Davidson, 2003); higher scores better), and experience of positive (six-question construct; higher scores better) and negative (five-question construct; lower scores better) emotions. The experience of emotions question set is informed by the positive psychology broaden-and-build theory (Fredrickson, 2001) and is adapted from the affective circumplex (the mapping of different emotional states into the dimensions of arousal and valence (Barrett and Russell, 1999, Watson and Tellegen, 1985), and the Affect Valuation Index developed by Tsai and Knutson (Tsai et al., 2006). The experience of emotions measure addresses different emotional states, including high arousal pleasant states (excitement, joy), neutral pleasant states (happy, content), low-arousal pleasant states (calm, secure), high arousal unpleasant states (worried, frustrated), neutral unpleasant states (sad), and low arousal unpleasant states (drained). Distress was assessed at T0, T1, and T2 (single item, National Comprehensive Cancer Network [NCCN] distress “thermometer” from 0 to 10 (Network et al., 2020); lower score better). Self-reported depression was also assessed as a binary outcome (single question adapted from the NCCN checklist (Network et al., 2020), measured at T0, T1, and T2). In addition to these psychosocial outcomes, full compliance with the SIP order was also considered as a binary outcome (single question: “I am staying at home nearly all the time; I only leave home to buy food and other essentials, to go to work (considered essential), or to exercise with social distance”; measured at T1 and T2), given its relevance to public health and relationship to psychological wellbeing. All constituent questions for each outcome variable are provided as Supplemental Material.

### 2.4 Covariates

Individual-level covariates derived from the WELL-baseline survey were included in fully-adjusted models (described below): age in years (continuous), gender, race, marital status, household income, and self-reported history of clinical depression. Household size during SIP (asked at T0) was also included.

### 2.5 Statistical analyses

Linear mixed-effects models were used to investigate the relationship between the continuous primary outcomes (resilience, stress, positive emotions, negative emotions, distress) and CPB (any CPB, average frequency of all CPB) across all available time points, and generalized linear mixed-effects models were used for binary primary outcomes (depression, SIP compliance). Random intercepts were included in models to account for repeated measures within participants. A continuous time variable (number of days from when SIP order took effect to participant’s response) was also included as a covariate in all models; for participants with W0 data collected less than a year prior to the COVID follow-up surveys, this SIP duration variable was set to zero. W0 data collected more than a year prior to the follow-up surveys were excluded, given the increasing possibility that changes would have occurred prior to the initiation of the COVID surveys. To further explore potential relationships between CPB and the outcome variables throughout the study period, the significance of interactions between a time and CPB was also considered by comparing models with and without this interaction term. Models with significant interaction terms were visualized using the *visreg* package (Breheny and Burchett, 2017).

To improve normal distributions of continuous outcome variables, Tukey’s Ladder of Powers was applied using the *transformTukey* function (Mangiafico, 2020). All continuous variables were scaled and centered by subtracting the mean and dividing by the standard deviation. All mixed-effects models were fit with the lme4 package in R using restricted maximum likelihood estimation (Bates, et al., 2020). Outliers were identified as observations with residuals greater than 2.5 standard deviations from the mean, and removed (see Supplemental Material) and models were re-fit without these observations. Conditional F-tests with Kenward-Roger approximations were used to compute p-values. All data processing and statistical modeling was performed using R statistical software.

## 3 Results

**Fig. 1:**
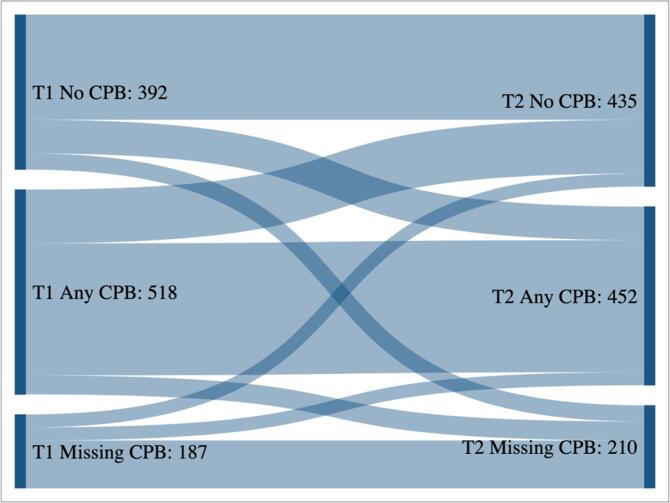
Diagram of Participant-Level Change in Any CPB between WELL Covid-19 Follow-Up Time 1 and Time 2.

Participants in this study were predominantly women (78.9%), with a mean age of 49.4 years (SD = 16.9). Most participants identified as white (64.8%), married or cohabiting (70.2%), and having a postgraduate or professional degree (48.4%) (see Table 1). Contemplative practices were fairly common at WELL-Baseline (W0) across the various demographic categories used in this study (see Table 1). Over half of the study population engaged in at least one contemplative practice at each of the three time points where CPB data were collected (57% at W0 [n = 619], 57% at T1 [n = 518], 51% at T2 [n = 452]). Compassion practices toward others had the highest average frequency, followed closely by non-reactive meditation practices (see Table 2). Approximately 32% of the study sample were consistently engaged with at least one contemplative practice at W0 and at both follow-ups (T1 and T2), and roughly a quarter of participants never reported engaging with contemplative practices. Fig. 1 illustrates participant-level changes in CPB between T1 and T2, with the majority of participants remaining consistent between the two periods, and smaller numbers adopting or discontinuing CPB.

**Table 1:** Selected Characteristics of Study Participants and contemplative practice behaviors at WELL-Baseline (W0) and Follow-Up (T1 and T2).

	W0 (2015–2020) n = 1085			T1 (04/03/20–4/12/2020) n = 847			T2 (04/23/20–5/7/2020) n = 887		
**Participant Characteristics**	*No CPB (n)*	*Any CPB (n)*	*Any CPB (%)*	*No CPB (n)*	*Any CPB (n)*	*Any CPB (%)*	*No CPB (n)*	*Any CPB (n)*	*Any CPB (%)*
Number of responses	467	618	57%	329	518	61%	435	452	51%

**Age at first follow-up (T0)**									
18–30	83	99	54%	75	73	49%	72	61	46%
31–40	107	107	50%	81	97	54%	99	68	41%
41–50	69	98	59%	57	76	57%	65	70	52%
51–60	79	129	62%	73	102	58%	73	104	59%
61–70	62	108	64%	56	96	63%	66	85	56%
71 and older	67	77	53%	50	74	60%	60	64	52%

**Gender**									
Female	357	498	58%	306	418	58%	335	363	52%
Male	105	113	52%	80	96	55%	96	85	47%
Trans/Other gender	5	3	38%	4	2	33%	3	2	40%

**Race**									
White/Caucasian	307	395	56%	258	341	57%	288	305	51%
Asian or Pacific Islander	105	147	58%	85	115	58%	92	97	51%
Black/African American	8	11	58%	4	12	75%	4	11	73%
Multiracial/Other Race	43	53	55%	39	44	53%	44	34	44%

**Educational attainment**									
High school and under	9	16	64%	7	13	65%	7	14	67%
Some college/Associate/Technical degree	45	54	55%	34	49	59%	37	36	49%
Bachelors/university level	167	258	61%	168	189	53%	176	161	48%
Post-graduate/professional	244	283	54%	180	263	59%	212	237	53%

**Household annual income (US $)**									
<$50,000	37	53	59%	28	52	65%	31	40	56%
$50,000 - $74,999	47	82	64%	50	58	54%	57	47	45%
$75,000 - $99,999	61	74	55%	50	59	54%	56	52	48%
$100,000 - $149,999	91	145	61%	79	121	61%	90	104	54%
$150,000 - $249,999	119	142	54%	106	112	51%	113	106	48%
$250,000 or more	98	99	50%	68	97	59%	74	85	53%

**Marital status**									
Married or cohabiting	326	435	57%	268	365	58%	299	316	51%
Single	95	109	53%	83	91	52%	89	79	47%
Other	46	74	62%	41	62	60%	47	57	55%

**Self-report of clinical depression at WELL-Baseline (W0)**									
No history of depression	360	469	57%	305	386	56%	338	342	50%
History of depression	107	145	58%	87	129	60%	97	107	52%

**Household size during SIP**									
1	64	77	55%	57	65	53%	59	66	53%
2	200	254	56%	150	232	61%	165	205	55%
3	86	113	57%	71	91	56%	83	75	47%
4	70	106	60%	71	81	53%	72	73	50%
5 or more	46	63	58%	43	48	53%	56	30	35%

**Years in WELL registry**									
<1	56	82	59%	56	66	54%	71	48	40%
1	134	171	56%	105	147	58%	114	119	51%
2	184	247	57%	146	211	59%	160	195	55%
3	49	63	56%	38	55	59%	40	51	56%
4	44	55	56%	47	39	45%	50	39	44%

**Table 2:** Descriptive Summary of Contemplative Practices Behaviors (W0, T1, T2).

	W0 (2015–2020)		T1 (04/03/20–4/12/2020)		T2 (04/23/20–5/7/2020)	
	*mean*	*sd*	*mean*	*sd*	*mean*	*sd*
*Frequency of contemplative practices*						
Non-reactive meditation practices a	2.2	1.1	2.1	1.2	2.1	1.2
Embodied-observing meditation practices a	1.8	1.2	1.8	1.2	1.7	1.3
Self-compassion practices a	1.9	1.0	1.8	1.1	1.7	1.1
Compassion practices toward others a	2.3	1.0	2.2	1.1	2.1	1.1
*Contemplative practice behaviors (CPB)*^b^	2.0	0.9	2.0	1.0	1.9	1.0

a Measured on ordinal scale 0–4.

b Measured as average of four contemplative practices.

**Fig. 2:**
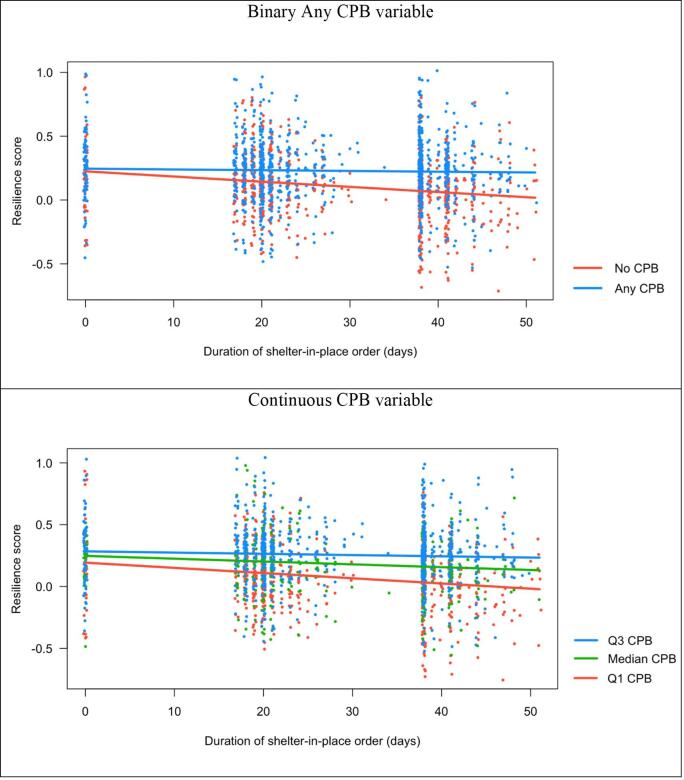
Significant interactions between CPB and days under SIP order for resilience outcome.

**Fig. 3:**
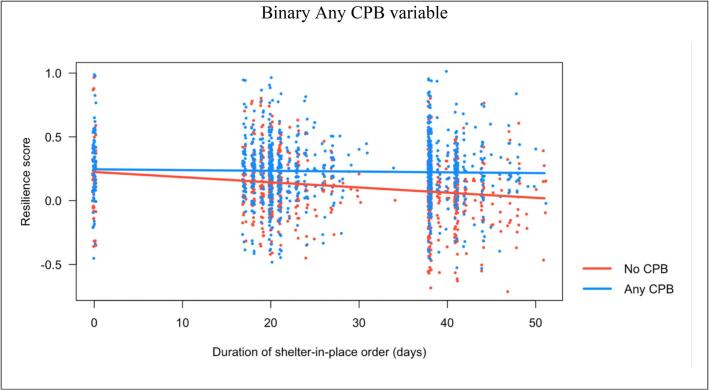
Significant interactions between CPB and days under SIP order for dealing with stress outcome.

**Fig. 4:**
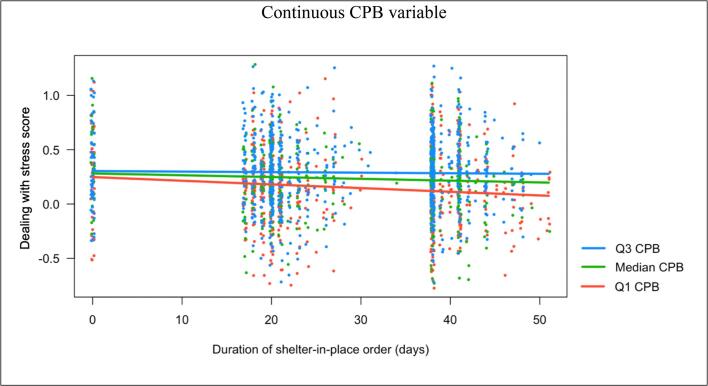
Significant interactions between Any CPB and days under SIP order for SIP compliance outcome.

In mixed-effects models, participants who reported any CPB had significantly better scores for dealing with stress (95% CI 0.01,0.16), experience of positive emotion (CI 0.15,0.28), and were significantly less likely to report experiencing depression in the last week (CI −0.04, −0.02). Relationships of a similar magnitude were found regarding the frequency of CPB (see Table 3), with the addition of higher CPB being significantly associated with less experience of negative emotion (CI −0.11, −0.02), and less distress (CI −0.10, −0.00). Significant interactions between CPB and the duration of the SIP order were observed in models for resilience (both binary and continuous CPB variable models, p = 0.036 and p = 0.003, respectively), perceived stress (continuous CPB variable model, p = 0.039) and SIP compliance (binary CPB variable model, p = 0.016). Fig. 2 illustrates how participants reporting any CPB were not as likely to experience declines in resilience (Fig. 2, top frame), and how this relationship was more pronounced by at higher average levels of CPB (Fig. 2, bottom frame). Similarly, the positive relationship between better perceived stress outcomes and CPB was greater at higher levels of CPB (see Fig. 3). In terms of full SIP compliance, average declines were observed over time for all participants, but this decline was significantly steeper among those without any CPB (see Fig. 4).

**Table 3:** Relationship between CPB variables and primary outcomes in mixed effects models.

		Any CPB (Yes)	Amount of CPB
*Continuous Outcomes (linear mixed-effects models)*			
Resilience ^a^	Estimate	–	–
	95% CI	–	–
	adjusted p-value	–	–

Dealing with Stress ^a^	Estimate	0.08	–
	95% CI	0.01–0.16	–
	p-value	0.019	–

Distress	Estimate	−0.01	−0.05
	95% CI	−0.10–0.07	−0.10 to −0.00
	p-value	0.778	0.048

Positive Emotions	Estimate	0.22	0.20
	95% CI	0.15–0.28	0.16–0.24
	p-value	<0.001	<0.001

Negative Emotions	Estimate	−0.02	−0.06
	95% CI	−0.09–0.05	−0.11 to −0.02
	p-value	0.514	0.003

*Binary Outcomes (generalized linear mixed-effects models)*			
Depression	Estimate	−0.03	−0.01
	95% CI	−0.04 to −0.02	−0.02 to −0.01
	p-value	<0.001	<0.001

SIP Compliance ^a^	Estimate	–	0.01
	95% CI	–	−0.01–0.03
	p-value	–	0.204

*Notes:*^a^ Given significant interactions between CPB variable and time, main CPB effects not reported here; Adjusted for participant age (continuous), gender, race, marital status, educational attainment, self-reported history of clinical depression at W0 baseline, annual household income and size during SIP (T0).

## 4 Discussion

In this study with repeated measurements we found positive relationships between contemplative practices and dealing with stress, and lower distress and depression, despite the multifaceted demands of the COVID-19 pandemic, which have been shown to produce adverse psychological outcomes in populations across the world (El-Hage et al., 2020, Huang and Zhao, 2020, Marton et al., 2020, Rossell et al., 2021, Daly et al., 2021, Charles et al., 2021, Castellano-Tejedor et al., 2021, Luo et al., 2020). These results suggest that CPB may play a salutogenic role and promote health in the face of adversity, although the specific mechanisms are unclear. Our findings also suggest that having any CPB is better than none for most of the psychosocial outcomes evaluated. While we find that more frequent CPB is positively associated with a variety of outcomes, results of a similar magnitude were obtained when examining the association between *any* CPB practice and these same outcomes. Notably, the resilience outcome appears to be a possible exception to this pattern, as the interaction effect observed suggests that people with higher levels of CPB experienced significantly smaller declines in resilience across the study period.

Studies of contemplative practices in neuroscience have provided further biological and behavioral insight into the relationships observed here (Eriksen and Ditrich, 2015). Data from other studies also suggest a possible relationship between CPB and better physical health, emotions, immune function and social connections (Fredrickson et al., 2013, Kok et al., 2013). Similarly, research on stress, especially one’s ability to positively cope with stressful situations and circumstances, also indicates downstream physical benefits (Thoits, 2010). Thus, it is possible that the emotion-related patterns observed here could lead to other health outcomes with direct relevance to individual susceptibility and vulnerability to both COVID-19 and the development of chronic diseases.

Psychobiological and behavioral studies have also shown that contemplative practices develop one’s capacity to acknowledge impermanence, and calmly and compassionately observe external and internal phenomena (Charney, 2004). These include a range of experiences, from internal body sensations thoughts and emotions to external economic uncertainty, which, in particular, has been found to increase globally amidst the COVID-19 pandemic (Baker et al., 2020). Thus, behavioral strategies that promote individuals’ ability to positively cope with impermanence and uncertainty are urgently needed at present, though their applicability transcends the unique challenges presented by COVID-19.

Though they are considered to be essential to reducing transmission of COVID-19, new investigations have shown how strict social distancing measures are associated with negative mental health outcomes. Quarantine measures in Italy have been attributed to higher levels of anxiety and loneliness (Barari et al., 2020), and local “lockdown” orders in Germany were associated with a 20 percent rise in mental health hotline contacts (Armbruster and Klotzbücher, 2020). Other studies suggest that greater support for quarantined populations could be warranted to mitigate the adverse psychological effects of social isolation. A Canadian study found that periods of self-insolation resulting from experiencing COVID-19 symptoms or possible viral exposure were associated with higher suicidal ideation compared to the non-isolating population (Daly et al., 2021), and evidence from Spain indicates that individuals with pre-existing vulnerabilities were most likely to suffer negative psychological impacts during COVID-19 confinement (Castellano-Tejedor et al., 2021). Other studies from the United States provide insights into how and why individuals comply with social distancing and “lockdown” policies, with some identifying capacity to follow distancing orders as well as personal self-control as key predictors of compliance (Andersen, 2020, van Rooij et al., 2020). Here, we found that participants who reported any CPB during the study period were more likely to report full compliance with a local shelter-in-place order than those with reporting no CPB. Further investigations are needed to unpack this relationship, though plausible explanations might involve the potential of CPB for developing and enhancing individuals’ adaptability, coping and acceptance and commitment cognitive behavioral self-control capabilities (Eifert et al., 2005).

### 4.1 Implications for practice

Our findings suggest the potential utility of CPB to help individuals cope with the psychological strains and behavioral demands of shelter-in-place orders related to the pandemic. To date, many grassroots efforts have arisen to provide free contemplative practice resources to the public during the pandemic, including Stanford Medicine’s web-based “Shelter-in-PEACE” program. Similarly, several commercially-provided apps such as “Headspace” and “10% Happier,” motivated by evidence from clinical observational and intervention outcomes, have offered free subscriptions to health care workers, educators and other essential workers, though evaluations have yet to be published. While these and other popular efforts to promote the multifaceted benefits of contemplative practices appear to be increasing, especially in light of the ubiquitous stressors emanating from the COVID-19 pandemic, further work is needed to fully incorporate these behaviors into the broader cadre of recommended health behaviors, such as those related to diet, physical activity, and sleep.

Innovative suggestions for public health measures to promote contemplative practices as an effective “emotional health hygiene” practice might be considered alongside the now-commonplace recommendations for handwashing hygiene, to be protective against the psychological and emotional ill health that can accompany a global viral pandemic. Given the ongoing disruptive nature of COVID-19, these recommendations might be used to address individuals’ current mental and emotional health needs, but also could help build healthy capacity for coping with future disruptions, including economic strains related to the pandemic disparities made visible by the pandemic, and possible re-issuances of SIP orders should COVID-19 re-escalate. Beyond the potential for helping individuals cope with their present circumstances and build resilience against future challenges, researchers also suggest that contemplative practices could be useful in addressing trauma stemming from COVID-19 related incidents (Chopko and Schwartz, 2009).

### 4.2 Strengths and limitations

Though not specifically designed to examine the causal effects of CPB on psychosocial outcomes (e.g., as in a randomized controlled trial), we leverage a longitudinal dataset that includes participants who move between doing any and no CPB, as well as between lower and higher levels of CPB during the study period. Thus, we are able to roughly approximate a treatment effect by modeling interactions between CPB variables and time, and find significant effects for resilience, perceived stress, depression and SIP compliance, all in directions suggesting a beneficial effect of CPB. With additional follow-up, these patterns and exposures can be further explored, especially as we come to better understand the characteristics of the participants who have adopted some kind of CPB during the COVID-19 pandemic.

This study has several limitations. As has been described elsewhere, participants in the WELL registry have, on average, relatively high incomes and education levels (see Table 1); though this is, in part, reflective of the San Francisco Bay Area, but not the general population. Additionally, though gender is relatively balanced in the baseline WELL registry, women are far more represented in the COVID-19 surveys. Our models were adjusted for these and other sociodemographic characteristics, and the reported CPB associations remained significant after adjustment. Furthermore, the prevalence of CPB is fairly consistent across a variety of demographic variables in the WELL registry, and participants were not purposively recruited based on CPB experience. Still, while we can report on the significance of associations within our study sample, some caution is needed on the generalizability of these findings to other settings.

Another limitation originates in the different dates of entry into the WELL registry, with some participants enrolling in 2015 and others as recent as 2020. While we excluded W0 observations that were more than a year old, the included observations are only an approximate baseline for the CPB variables and outcomes and are not as temporally comparable as those collected in the COVID-19 follow-up surveys. Thus, it is possible that changes to health and personal circumstances may have a differential influence due to the length of time since the change was measured. Further planned follow-up surveys will allow for more detailed examination of the longitudinal patterns described in this analysis, which could also help understand if and how other coping behaviors changed alongside CPB during the pandemic. With additional data collected in follow ups, we aim to investigate the roles of CPB in relation to alcohol, tobacco and cannabis use, physical activity, resilience, and the quality of interpersonal relationships, which could be correlated with CPB. This study offers a high-level summary of overall associations with relevant psychosocial outcomes, with additional investigations needed to understand the underlying mechanisms at work.

A final limitation is related to the depression measure, which is a single-item from the NCCN Distress Thermometer. While we include a history of clinically diagnosed depression as a covariate, we recognize that the limitations of this outcome measure versus more comprehensive tools. Other research suggests that single-item self-reports of depression may still offer useful screening mechanisms in advance of more detailed questionnaires, such as the Hospital Anxiety and Depression Scale (HADS) (Turon et al., 2019, Zigmond and Snaith, 1983).

### 4.3 Conclusion

4.3

The positive associations between CPB and positive mental and emotional health outcomes, and SIP compliance merit further investigation and consideration by public health officials dealing with the COVID-19 pandemic. Contemplative practices are a form of affordable and accessible disaster and pandemic preparedness that could be broadly offered as a public health behavioral skill for sustaining health and well-being, comparable to the skills of hand washing or mask wearing, and play a part in assisting people who are exposed to trauma directly or vicariously. Inclusion of contemplative practice behaviors as part of public health guidelines for healthy lifestyle recommendations for the non-clinical population have the potential of enhancing resilience, posttraumatic growth, physical and emotional health, and offer a promising approach to reducing distress and depression.

## CRediT authorship contribution statement

**Benjamin W. Chrisinger:** Conceptualization, Methodology, Writing - review & editing, Investigation, Writing - original draft, Formal analysis. **Tia Rich:** Conceptualization, Writing - original draft, Writing - review & editing. **David Lounsbury:** Writing - review & editing. **Katy Peng:** Data curation. **Janice Zhang:** Data curation. **Catherine A. Heaney:** Writing - review & editing. **Ying Lu:** Writing - review & editing. **Ann W. Hsing:** Funding acquisition, Writing - review & editing, Project administration.

## Declaration of Competing Interest

The authors declare that they have no known competing financial interests or personal relationships that could have appeared to influence the work reported in this paper.
