# Supplementary material for: Coping with the COVID-19 pandemic: Contemplative practice behaviors are associated with better mental health outcomes and compliance with shelter-in-place orders in a prospective cohort study
Source: Prev Med Rep. 2021 Jun 12;23:101451. doi: 10.1016/j.pmedr.2021.101451 (PMC8220389; doi:10.1016/j.pmedr.2021.101451)
Supplement: Supplementary data 1 [file mmc1.docx]

**Supplemental Table 1**. Contemplative Practice Variables Calculated at Each Time Point

| **Variable** | **Definition** | **Variable structure** |
| --- | --- | --- |
| 1. Any contemplative practices | Responded sometimes, fairly often, or very often for any of 4 CPs | Binary (Yes/No) |
| 1. Amount of contemplative practice behaviors (CPB) | Average frequency (0-4, never to very often) of 4 CPs | Continuous (0-4) |
| **Survey items about the frequency of contemplative practices** | | |
| *Select one: a. Very often, b. Fairly often, c. Sometimes, d. Almost never, e. Never* | | |
| 1. During the last two weeks, how often did you take at least five minutes to pause your routine activities to quiet your thinking mind, for example by breathing deeply, gently stretching, noticing your senses, or simply resting?  2. During the last two weeks, how often did you take at least five minutes to pause your routine activities to meditate, by observing emotions and thoughts as they arise, rather than being caught up in them?  3. During the last two weeks, how often did you take at least five minutes to pause your routine activities to observe and modify the way you were thinking in order to offer more compassion, love or kindness to others?  4. During the last two weeks, how often did you take at least five minutes to pause your routine activities to observe and modify the way you were thinking to offer more compassion, love or kindness to yourself?” | | |

**Supplemental Table 2**. Summary of Outliers Removed from Final Statistical Models

| Independent Variable | Dependent Variable | N removed | % removed | Interaction Model |
| --- | --- | --- | --- | --- |
| Any CPB | Resilience | 48 | 2.6 | Y |
|  | Dealing with Stress | 40 | 2.2 | N |
|  | Distress | 25 | 1.5 | N |
|  | Positive Emotions | 32 | 1.7 | N |
|  | Negative Emotions | 37 | 2.0 | N |
|  | Depression | 99 | 5.8 | N |
|  | SIP Compliance | 67 | 4.0 | Y |
| CPB | Resilience | 48 | 2.6 | Y |
|  | Dealing with Stress | 41 | 2.2 | Y |
|  | Distress | 26 | 1.5 | N |
|  | Positive Emotions | 33 | 1.8 | N |
|  | Negative Emotions | 37 | 2.0 | N |
|  | Depression | 94 | 5.5 | N |
|  | SIP Compliance | 72 | 4.3 | N |

**CODING FOR CONTEMPLATIVE PRACTICES**

Measured at WELL-Baseline; COVID-19 Study T1, T2 (used as primary IV)

- The frequency of practice was measured on an ordinal scale (0-5: Never, Almost never, Sometimes, Fairly often, Very often).
- Practice of mindfulness:
  - embodied-observing practices (breathing deeply, gently stretching, noticing your senses)
  - non-reactive practices (observing emotions and thoughts as they arise rather than being caught up in them).
- Compassion practice:
  - self-compassion practice (pausing routine activities to observe and modify the way one is thinking to offer more compassion, love or kindness to oneself)
  - compassion practice toward others (pausing routine activities to observe and modify the way one is thinking to offer more compassion, love or kindness toward others).

**CODING FOR DEPRESSION**

WELL-Baseline (used as covariate)

- Medical diagnosis: Depression (Yes/No)

COVID-19 Study T1, T2 (used as DV)

- [Adapted from National Comprehensive Cancer Network Distress Thermometer and Problem List: <https://www.nccn.org/patients/resources/life_with_cancer/pdf/nccn_distress_thermometer.pdf> ] Please indicate if any of the following has been a problem for you in the past week including today: Depression (Yes/No)

**CODING FOR SHELTER-IN-PLACE COMPLIANT**

COVID-19 Study T0, T1, T2 (used as DV)

- Have you been compliant with the Shelter-in-Place order? “All of the time. I am staying at home nearly all the time; I only leave home to buy food and other essentials, to go to work (considered essential), or to exercise with social distance”).

**CODING FOR DISTRESS**

COVID-19 Study T0, T1, T2 (used as DV)

- [Adapted from National Comprehensive Cancer Network Distress Thermometer and Problem List: <https://www.nccn.org/patients/resources/life_with_cancer/pdf/nccn_distress_thermometer.pdf> ] Please indicate if any of the following has been a problem for you in the past week including today (Yes/No):
  - depression
  - fears
  - nervousness
  - sadness
  - worry
  - Loss of interest in usual activities
  - fatigue
  - memory/concentration
  - sleep
- Distress score is the total number of a participant’s “Yes” responses at a given time point.

**CODING FOR STRESS and RESILIENCE**

COVID-19 Study T0, T1, T2

Stress items are noted in Red = #10, 11, 12, 13, 14

Resilience items are noted in Blue = #1, 2, 3, 4, 5, 6, 7, 8, 9

**Domain:** Stress and Resilience

**Definition:** Resilience is described as the ability to/experience of adapting to change or tendency to bounce back after illness or hardship, ability to effectively manage stress, ability to balance tasks. Stress is described as a feeling of overload, being overwhelmed, out of control, using the term “stress," inability to balance or manage tasks.

**Sources of Items:**

Cohen, S., Kamarck, T., Mermelstein, R. (1983). A global measure of perceived stress. *Journal of Health and Social Behavior,* *24*, 385-396. Retrieved from http://www.psy.cmu.edu/~scohen/globalmeas83.pdf. [Perceived Stress Test (PSS)]

Connor, K. M., & Davidson, J. R. T. (2003). Development of a new resilience scale: The Connor-Davidson Resilience scale (CD-RISC). *Depression and Anxiety, 18*(2), 76–82. <http://doi.org/10.1002/da.10113> [CD-RISC]

Smith, B. W., Dalen, J., Wiggins, K., Tooley, E., Christopher, P., & Bernard, J. (2008). The brief resilience scale: assessing the ability to bounce back. *International journal of behavioral medicine*, *15*(3), 194-200. doi: 10.1080/10705500802222972 [Brief Resilience Scale (BRS)]

WELL Measures Workgroup, 2016 [WELL]

**Items:**

1. [Adapted from BRS] How confident are you that you can bounce back quickly after hard times? 5, Extremely confident | 4, Very confident | 3, Moderately confident | 2, Slightly confident | 1, Not at all confident

[Original question from BRS] Item 1. I tend to bounce back quickly after hard times. Strongly Agree | disagree | neutral | agree | strongly agree

1. [Adapted from CD-RISC] How confident are you that you can adapt to change? 5, Extremely confident | 4, Very confident | 3, Moderately confident | 2, Slightly confident | 1, Not at all confident

[Original question from CD-RISC] Item V1. I am able to adapt to change. Rated from "not true at all" to "true nearly all the time".

1. [Adapted from CD-RISC] How confident are you that you can deal with whatever comes your way? 5, Extremely confident | 4, Very confident | 3, Moderately confident | 2, Slightly confident | 1, Not at all confident

[Original question from CD-RISC] Item V4. I can deal with whatever comes my way. Rated from "not true at all" to "true nearly all the time".

1. [Adapted from CD-RISC] How confident are you that you can see the humorous side of problems? 5, Extremely confident | 4, Very confident | 3, Moderately confident | 2, Slightly confident | 1, Not at all confident

[Original question from CD-RISC] Item V6. I see the humorous side of things. Rated from "not true at all" to "true nearly all the time".

1. [WELL] How confident are you that you can overcome obstacles? 5, Extremely confident | 4, Very confident | 3, Moderately confident | 2, Slightly confident | 1, Not at all confident
2. [Adapted from CD-RISC] How confident are you that you can stay focused under pressure? 5, Extremely confident | 4, Very confident | 3, Moderately confident | 2, Slightly confident | 1, Not at all confident

[Original question from CD-RISC] Item V14. Under pressure, I can focus and think clearly. Rated from "not true at all" to "true nearly all the time".

1. [Adapted from CD-RISC] How confident are you that you can think of yourself as a strong and resilient person? 5, Extremely confident | 4, Very confident | 3, Moderately confident | 2, Slightly confident | 1, Not at all confident

[Original question from CD-RISC] Item V17. I think of myself as a strong person. Rated from "not true at all" to "true nearly all the time".

1. [Adapted from CD-RISC] How confident are you that you can manage any unpleasant feelings that you might have? 5, Extremely confident | 4, Very confident | 3, Moderately confident | 2, Slightly confident | 1, Not at all confident

[Original question from CD-RISC] Item V19. I can handle unpleasant feelings. Rated from "not true at all" to "true nearly all the time".

1. [Adapted from CD-RISC] How confident are you that you can not get disheartened by setbacks? 5, Extremely confident | 4, Very confident | 3, Moderately confident | 2, Slightly confident | 1, Not at all confident

[Original question from CD-RISC] Item V16. I am not easily discouraged by failure. Rated from "not true at all" to "true nearly all the time".

1. [Adapted from PSS] During the last two weeks, how often have you felt that you were not able to give enough time to the important things in your life? 5, Very often | 4, Fairly often | 3, Sometimes | 2, Almost never | 1, Never

[Original question from PSS] Item 2. In the last month, how often have you felt that you were unable to control the important things in your life? Never | Almost never | Sometimes | Fairly often | Very often

1. [Adapted from PSS] During the last two weeks, how often have you felt that you were able to handle the problems you are experiencing? 5, Very often | 4, Fairly often | 3, Sometimes | 2, Almost never | 1, Never

[Original question from PSS] Item 6. In the last month, how often have you felt confident about your ability to handle your personal problems? Never | Almost never | Sometimes | Fairly often | Very often

1. [Adapted from PSS] During the last two weeks, how often have you felt that things were going your way? 5, Very often | 4, Fairly often | 3, Sometimes | 2, Almost never | 1, Never

[Original question from PSS] Item 7.  In the last month, how often have you felt that things were going your way? Never | Almost never | Sometimes | Fairly often | Very often

1. [Adapted from PSS] During the last two weeks, how often have you felt overwhelmed by difficulties in your life? 5, Very often | 4, Fairly often | 3, Sometimes | 2, Almost never | 1, Never

[Original question from PSS] Item 14.  In the last month, how often have you felt difficulties were piling up so high that you could not overcome them? Never | Almost never | Sometimes | Fairly often | Very often

1. [Adapted from PSS] During the last two weeks, how often have you felt that you were not able to give enough energy to the important things in your life? 5, Very often | 4, Fairly often | 3, Sometimes | 2, Almost never | 1, Never

[Original question from PSS] Item 2. In the last month, how often have you felt that you were unable to control the important things in your life? Never | Almost never | Sometimes | Fairly often | Very often
